# Definition and severity grading of postoperative lymphatic leakage following inguinal lymph node dissection

**DOI:** 10.1007/s00423-020-01927-7

**Published:** 2020-08-20

**Authors:** Andreas Lutz Heinrich Gerken, Florian Herrle, Jens Jakob, Christel Weiß, Nuh N. Rahbari, Kai Nowak, Constantin Karthein, Peter Hohenberger, Jürgen Weitz, Christoph Reißfelder, Jakob C. Dobroschke

**Affiliations:** 1 Department of Surgery, Universitätsmedizin Mannheim, Medical Faculty Mannheim, Heidelberg University, Theodor-Kutzer-Ufer 1-3, 68167 Mannheim, Germany; 2 Department of General, Visceral and Pediatric Surgery, University of Göttingen, Robert-Koch-Str. 40, 37075 Göttingen, Germany; 3 Department of Biometry and Statistics, University Medical Center Mannheim, University of Heidelberg, Theodor-Kutzer-Ufer 1-3, 68167 Mannheim, Germany; 4 Department of General Vascular and Thoracic Surgery, RoMed Hospital Rosenheim, Pettenkoferstraße 10, 83022 Rosenheim, Germany; 5 Department of Visceral Surgery, University Hospital, Technical University Dresden, Fetscherstr. 74, 01307 Dresden, Germany

**Keywords:** ILND, Complications, Outcome, Lymphogenic morbidity, Lymphatic fistula, Melanoma

## Abstract

**Purpose:**

Lymphatic complications occur frequently after radical inguinal lymph node dissection (RILND). The incidence of lymphatic leakage varies considerably among different studies due to the lack of a consistent definition. The aim of the present study is to propose a standardized definition and grading of different types of lymphatic leakage after groin dissection.

**Methods:**

A bicentric retrospective analysis of 82 patients who had undergone RILND was conducted. A classification of postoperative lymphatic leakage was developed on the basis of the daily drainage output, any necessary postoperative interventions and reoperations, and any delay in adjuvant treatment.

**Results:**

In the majority of cases, RILND was performed in patients with inguinal metastases of malignant melanoma (*n* = 71). Reinterventions were necessary in 15% of the patients and reoperations in 32%. A new classification of postoperative lymphatic leakage was developed. According to this definition, grade A lymphatic leakage (continued secretion of lymphatic fluid from the surgical drains without further complications) occurred in 13% of the patients, grade B lymphatic leakage (persistent drainage for more than 10 postoperative days or the occurrence of a seroma after the initial removal of the drain that requires an intervention) in 28%, and grade C lymphatic leakage (causing a reoperation or a subsequent conflict with medical measures) in 33%. The drainage volume on the second postoperative day was a suitable predictor for a complicated lymphatic leakage (grades B and C) with a cutoff of 110 ml.

**Conclusion:**

The proposed definition is clinically relevant, is easy to employ, and may serve as the definition of a standardized endpoint for the assessment of lymphatic morbidity after RILND in future studies.

## Introduction

Radical inguinal lymph node dissection (RILND) is performed in patients with inguinal metastases of malignant diseases of the lower extremities or of the genital or anal regions. Wound complications occur frequently after RILND, reported to range from 14 to 77% in published studies [1–6]. The high rate of wound complications can be explained by the postoperative collection of fluid in the wound caused by the inevitable transection of lymphatic vessels that failed to agglutinate. Suction drains are usually placed in the wound after resection in order to prevent the accumulation of fluid within the resection cavity and to promote the adherence of the wound surfaces. However, in a large proportion of patients, the drains cannot be removed timely due to the persistent discharge of large amounts of lymphatic fluid. This condition is commonly described by the terms “lymphatic leakage,” “lymphatic fistula,” “seroma,” or “lymphocele.”

There is no standard method to classify persistent lymphatic leakage following RILND. Physicians therefore use empirical measures to plan subsequent treatment. Our group reasons that a standardized classification would provide valuable information to guide further treatment. A systematic review and meta-analysis evaluating the benefit of applying tissue sealants to reduce postoperative lymphatic leakage after RILND in melanoma patients that was recently published by our study group points out that it is difficult to conduct a valid comparison of the results of the trials studied due to inhomogeneous endpoint definitions [7]. The studies that were included used different endpoints to describe lymphogenic morbidity. The incidence of postoperative lymphatic leakage was not recorded, or its definition varied considerably among these studies. A definition of the term lymphatic leakage validated by patient data is required in order to standardize the reporting of outcomes and to compare the results of future trials consistently.

The aim of the present study is to introduce a simple and clinically applicable standardized definition for lymphatic leakage after groin dissection, which is actually lacking in literature. This definition should primarily serve as an indicator for the attribution of patients at risk for a complicated wound situation. Secondly, this definition could be used as a standardized endpoint definition for further research.

## Material and methods

### Patients and study design

All the patients scheduled for an RILND because of inguinal metastases of malignant diseases who underwent surgery between April 2009 and July 2014 in the University Hospital of Technical University Dresden and between January 2011 and March 2014 in the Mannheim University Medical Center were consecutively included into this retrospective study. Patients with superficial lymph node excisions or sentinel node biopsy were excluded. The study was conducted in congruence with the Declaration of Helsinki and further approved by the local ethics committees of the Universities of Mannheim and Dresden.

### Surgical procedure

Preoperative diagnostics and the surgical procedure adhered to the standard techniques. RILND was performed according to international standards (the NCCN clinical practice guidelines for melanoma). Monopolar and bipolar diathermy were used. No ultrasonic dissection was performed. A 12-French suction drain (Redon drain, e.g. ORIFLEX-4600 ml, Oriplast Krayer GmbH, Neunkirchen, Germany) was inserted into the resection cavity before wound closure. Wound closure was performed with a subcutaneous suture in single-stitch technique. The cutis was closed by single stitches in the back-stitch technique or with metal clips.

### Acquisition of Data

Electronic databases and patient files were used for data extraction. Assessed were general patient data, the daily postoperative drainage output after RILND, the incidence of wound complications or wound infection, the need for postoperative interventions like needle aspirations and reoperations, and the planned and actual dates of adjuvant treatment.

### Grading of lymphatic leakage

The severity of lymphatic leakage was graded according to clinically relevant considerations. Patients were assigned to one of the following four categories of severity.

Grade A lymphatic leakage was characterized by a prolonged secretion of lymphatic fluid from the drains inserted intraoperatively or the appearance of a seroma without the occurrence of wound complications, such as infection or wound breakdown, requiring reinterventions or reoperations. A “prolonged secretion” of lymphatic fluid was defined as a drainage output of at least 50 ml/24 h for more than 5 but less than 10 postoperative days.

Grade B lymphatic leakage was characterized by the prolonged leakage of lymphatic fluid from the surgically inserted drains of at least 50 ml/24 h for at least 10 postoperative days or by the appearance of seromas after an initial removal of the drains that required interventions.

Grade C lymphatic leakage was characterized by any condition of lymphatic leakage leading to a reoperation or causing any delay of further treatment (e.g., delay of planned adjuvant therapy as this is a quantifiable criterion in the retrospective setting showing a conflict with subsequent medical measures).

The remaining patients had no lymphatic leakage and were classified grade 0.

### Statistical analysis

All statistical calculations were performed with SAS, release 9.4 (SAS Institute Inc., Cary, North Carolina, USA). For qualitative data, absolute and relative frequencies were calculated. Quantitative results were represented by their median value together with the range. In order to compare two groups, chi^2^ test, Fisher’s exact test, or the Wilcoxon 2 sample test were used, as appropriate. Because of the rather small sample sizes, exact tests were performed. For the comparison of more than two groups regarding a quantitative variable (e.g., daily drainage volumes), the Kruskal-Wallis test was performed.

Logistic regression analysis was employed for testing the association between a binary outcome (e.g., leakage yes/no) and an explanatory quantitative variable (e.g., drainage volume). The area under the ROC curve (AUC) was assessed for quantifying the predictive ability of the model. Furthermore, the optimum cutoff point was estimated, which minimizes the Youden index (sensitivity + specificity – 1).

The result of a statistical test was considered statistically significant if the *p* value was less than 0.05.

## Results

### Description of the population

The population consisted of 82 patients who underwent RILND in two University Centers. Sixty-two resections were performed in the Department of Visceral Surgery of the University Hospital Dresden and twenty resections in the Department of Surgery of the University Medical Center Mannheim. The main indication for RILND was malignant melanoma. Other indications and further patient characteristics are presented in Table 1. There was a sentinel lymph node excision in 51 patients (62.20%) prior to the RILND. In 16 cases (19.51%), the RILND was extended by an iliac lymph node dissection. The great saphenous vein was resected in 39 (48%) operations. In 11 patients (13%), a medical therapy with Aspirin was recorded. Forty-three patients (52%) had low-molecular-weight heparin prior to the resection. The median albumin level was 43.70 g/l (range 32–52 g/l). The median number of resected lymph nodes was 9.0 (range 2–25), and the median number of infiltrated lymph nodes was 1.0 (range 0–15). The median operation time was 128 min (range 27–302 min).

**Table 1:** Patients’ characteristics, postoperative morbidity and grades of lymphatic leakage

**Patients** ***n*** **= 82**	**Median (range)**	**Postop. lymphatic leakage**	***n*** **(%)**
Sex (*n*: ♀:♂)	34:48	None*	21 (26)
Age (years)	60 (20–86)	Grade A	11 (13)
BMI (kg/m^2^)	27 (18–47)	Grade B	23 (28)
Obesity	22 (27)	Grade C	27 (33)
**RILND indications**	***n*** **(%)**	**Comorbidities**	***n*** **(%)**
Melanoma	71 (87)	Diabetes	13 (16)
Merkel cell carcinoma	4 (5)	Smoker	18 (27)
Others: S (*n* = 2), AC (*n* = 2), PC (*n* = 2), EC (*n* = 2), NHL (*n* = 1)	7 (8)		
**Operative and drainage details**	**Median (range)**	**Postoperative morbidity**	***n*** **(%)**
Duration of hospital stay	10 (4–79)	Seroma	29 (35)
Duration of surgery (min)	128 (27–302)	Needle aspiration	12 (15)
Duration of drainage (days)	7 (1–30)	Reoperations	26 (32)
Drainage volume day 2 (ml)	310 (45–1595)	Readmission	17 (21)
Drainage volume at removal (ml)	90 (0–1000)	Delay of adjuvant therapy	10 (12)

*AC* anal cancer, *EC* esophageal cancer, *NHL* Non-Hodgkin lymphoma, *PC* prostate cancer, *S* sarcoma

*None equals Grade 0

### Drain removal

Drainage details are presented in Table 1. In 15 cases, the drain was removed at an output of less than 50 ml/24 h. In 15 cases, the drainage volume at the time of removal was at least 50 ml/24 h but less than 100 ml/24 h, while in 25 patients, the output at the time of removal was 100 ml/24 h or more. The drainage volume at the time of removal was not reported for 21 patients. Six patients were discharged from the hospital with persistent suction drains. In 46 of all the patients (56%), the drain was removed within the first postoperative week, while in 12 patients (15%), the drain stayed in place for 3 weeks or longer.

### Daily postoperative drainage volumes

**Fig. 1:**
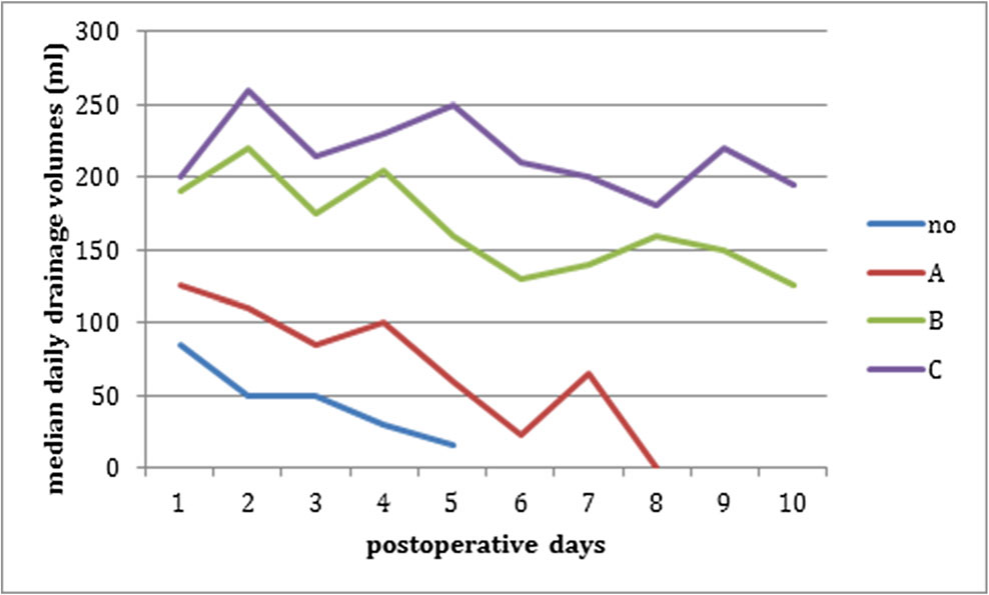
Course of median daily drainage volumes on postoperative days 1–10 in patients with different grades of lymphatic leakage (no = grade 0; A = grade A; B = grade B; C = grade C lymphatic leakage). Sample sizes for days 1 to 10: grade 0: *n* = 20, 18, 17, 10, 6, 3, 1, 0, 0, 0; grade A: *n* = 9, 10, 7, 9, 5, 6, 6, 1, 0, 0; grade B: *n* = 22, 23, 23, 22, 19, 17, 17, 15, 16, 13; grade C: *n* = 23, 23, 24, 20, 19, 18, 14, 11, 12, 12. Only medians values with *n* ≥ 5 are depicted

Figure 1 shows the median daily drainage volumes on postoperative days 1–10 in patients with different grades of lymphatic leakage. It demonstrates that higher drainage volumes correspond to higher grades of lymphatic leakage. Patients without a lymphatic leakage (grade 0) had a median drainage output of less than 50 ml/24 h after the second postoperative day. Patients with a grade A lymphatic leakage showed a persistent median drainage output of 50 ml/24 h or more during the first 5 postoperative days; however, a minor output on days 6 and 8. Grade B and C lymphatic leakage corresponded to a substantially larger amount of secretion of lymphatic fluid for more than 8 postoperative days.

On postoperative days 1–3, the median drainage volumes differed significantly between the different grades of lymphatic leakages (*p* = 0.0005 for day 1; *p* < 0.0001 for each day 2 and 3). Pairwise comparison tests revealed statistically significant differences for each day between grades 0 and B and between 0 and C (each *p* < 0.0010). Furthermore, significant differences between grades A and B were detected on days 2 and 3 (*p* = 0.0281 and *p* = 0.0485, respectively) as well as between A and C on days 2 and 3 (*p* = 0.0143 and 0.0405). Grades 0 and A differed significantly only on day 2 (*p* = 0.0317).

### Receiver operating characteristic

**Fig. 2:**
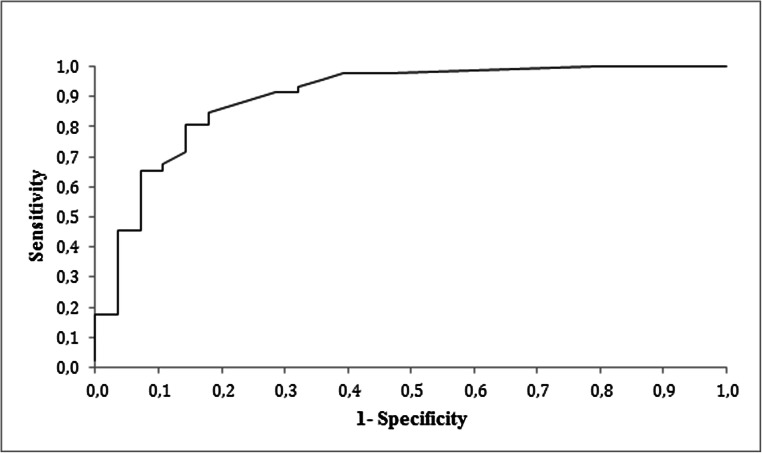
Receiver operating characteristic curve for the drainage volume on postoperative day 2 as a predictor for the occurrence of a complicated lymphatic leakage (grades B and C). AUC = 0.897, *p* < 0.0001

Logistic regression analysis testing the association of drainage volume and the binary outcome “lymphatic leakage grade B and C” versus “lymphatic leakage grade A and no lymphatic leakage” revealed that the AUC was 0.897 (*p* < 0.0001), representing a fairly good predictive value of the drainage volume. The optimum cutoff point in our curve was a drainage volume of 110 ml/24 h on postoperative day 2, delivering a sensitivity of 85% and a specificity of 82% for predicting a lymphatic leakage grade B or C (see Fig. 2).

### Lymphatic leakage-associated morbidity

Of the 82 patients, 17 (20.7%) were readmitted with complications. In 29 patients (35.4%), a seroma was detected, and 12 patients (14.6%) required a needle aspiration of the seroma. In 26 patients (31.7%), a reoperation had to be performed after a failure of conservative management, mainly vacuum-sealing procedures (*n* = 17) followed by surgical wound revision and reinsertion of a suction drain (*n* = 6), wound revision with closure of the leakage site (*n* = 2), and wound revision with secondary suture of the skin (*n* = 1). For 10 patients (12.2%), adjuvant therapy was delayed. The data is presented in Table 1.

### Variables predisposing to complicated lymphatic leakage

None of the following factors affected the incidence of a complicated lymphatic leakage grade B or C: smoking (*p* = 0.1662), diabetes (*p* = 0.1988), overweight (*p* = 0.4179), and preoperative therapy with low-molecular-weight heparin (*p* = 0.6862) or Aspirin (*p* = 1.0000). Other factors, however, such as the resection of the great saphenous vein (*p* = 0.0449), gender (*p* = 0.0571), or iliac lymph node resection (*p* = 0.0639) revealed test results being significant or close to being significant.

## Discussion

### Lymphatic complications after RILND

RILND is a surgical procedure with a substantially high incidence of wound complications, namely, up to 77% [1, 8]. The morbidity of RILND can be explained by the accumulation of lymphatic tissue in the inguinal region that is inevitably injured during RILND, leading in turn to lymphatic complications [9].

Different patient- and procedure-related risk factors for the occurrence of wound complications after RILND have been described in previous publications, such as smoking, diabetes, overweight, and long operating time [1, 2, 5, 10]. Interestingly, the perioperative administration of low-molecular-weight heparin has been identified as a risk factor for pelvic lymphoceles after radical prostatectomy [11].

To our knowledge, no guidelines on therapeutic approaches of lymphatic complications after RILND were consented upon as none of the available options has shown therapeutic efficiency in RCTs. A step-up approach from conservative treatment to intervention followed by reoperation has been suggested by Lv et al. [9]. Possible therapeutic interventions include the instillation of fibrin glue [12], talcum [13], doxycycline [14], erythromycin [15], Picibanil (OK-432) [16], or low-dose radiotherapy [17]. These interventions are performed with the aim to cause obliteration of leaking lymphatic channels allowing the adaptation of wound surfaces. Reoperations include ligation and suture of leaking lymphatic vessels, sealing with fibrin glue [18], and vacuum-sealing therapy [19].

Although the wound complication rate after RILND has been demonstrated to be reduced by different cointerventions or modifications of surgical technique, lymphogenic morbidity still remains high. In our study, 61% of the studied patients developed complicated wound situations after RILND. Furthermore, 12.2% experienced a delay in receiving adjuvant therapy due to wound complications. These results emphasize once again the relevant morbidity of RILND. Techniques aiming to reduce complications are for example prophylactic vacuum-sealing therapy [20], sartorius transposition [10, 21], or video-endoscopic minimally invasive inguinal lymphadenectomy (VEIL) [22] showing inconclusive results concerning the reduction of lymphatic leakage. Several previous RCTs have attempted to reduce lymphatic complications after RILND by employing hemostyptic agents, however, without using a consistent endpoint definition as confirmed by a current systematic review and meta-analysis [7]. A standardized definition of “postoperative lymphatic leakage after RILND” is necessary for any subsequent step to estimate the effect of prophylactic interventions to provide the patient with the best available operative technique and ultimately to achieve a better oncological outcome.

### Definition and grading of lymphatic leakage

Different endpoints in previous studies reflect several attempts to describe lymphogenic morbidity. These commonly used endpoints are for example duration of drainage, total drainage volume until removal of drains, or incidence of postoperative seroma. However, these endpoints depend on the definition of the drain removal criteria and might fail to register patients developing a complicated wound situation due to a seroma after an early drain removal. The range of definitions for lymphatic leakage starts from “leakage of lymph from the surgical wound” [3] to “continued discharge of lymphatic fluid to the skin surface” [19], “lymphatic secretion for more than 6 postoperative days” [23], or to persistent lymphatic leakage of more than 30 ml/day for more than 5 days [24]. Lv et al. deliver a more general classification of postoperative lymphatic leakage after a variety of different surgical procedures [9]; however, this definition remains unspecific for RILND.

**Fig. 3:**
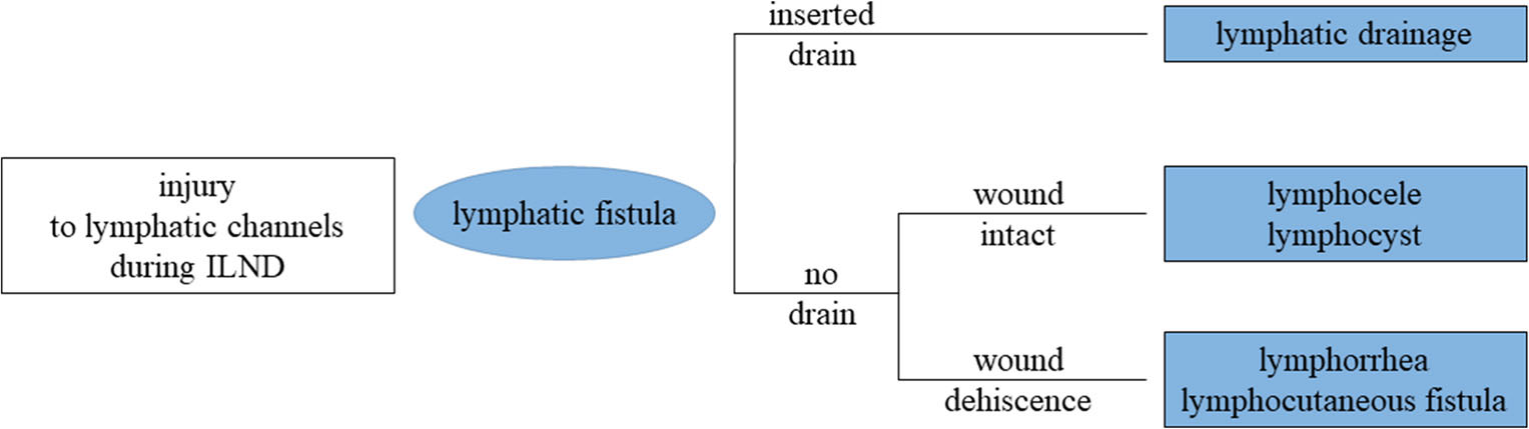
Definition of different types of lymphatic leakage after RILND

In adoption to Ly et al., we define three types of lymphatic leakage after RILND, as presented in Fig. 3: Firstly, “lymphatic drainage” as a special situation in which the wound is (still) drained by inserted drains preventing the accumulation of fluid in the wound cavity. Secondly, “postoperative seroma”/“lymphocele”/“lymphocyst” as accumulation of lymphatic fluid in the wound cavity given the integrity of the skin wound, diagnosed by any kind of imaging (US/CT/MRI) to document the existence and measure the amount of fluid accumulation—and also to differentiate it from hematoma, abscess, and other postoperative sequelae. And thirdly, “lymphorrhea”/“lymphocutaneous fistula” as secretion of lymphatic fluid through the surgical wound in case of dehiscence of wound margins, diagnosed clinically. A lymphatic fistula represents the underlying mechanism for the types of lymphatic leakage named above and is defined as leakage of lymphatic fluid due to unintended injury of lymphatic channels during surgery. It can be diagnosed by lymphoscintigraphy or lymphography.

The data in the present study demonstrate that lymphatic complications after RILND can be grouped into four distinct categories on the basis of the amount of daily drainage output and the need for interventions or reoperations. Table 2 shows our proposed definition of lymphatic leakage after RILND. We distinguish between situations in which a drainage tube is still inserted and a condition in which the drain has been removed. We suggest defining lymphatic leakage after RILND as a persistent secretion of lymphatic fluid of at least 50 ml/day from the surgically inserted drains or the wound for more than five postoperative days or postoperative fluid accumulation within the cavity of resection in the absence of drains. We also suggest a clinical grading system of lymphatic leakage, because the basic definition comprises all types of leakage ranging from asymptomatic leakage to a leakage resulting in severe discomfort or even wound breakdown or infection with the need for reoperation. The severity grading is based on the period of lymphatic secretion and the need for interventions or operations (Table 2). The introduction of measurable criteria is necessary for a standardized endpoint definition for further research, i.e., randomized trials of interventions.

**Table 2:** Proposal for the definition and severity grading of postoperative lymphatic leakage after radical inguinal lymph node dissection (RILND)

Definition of lymphatic leakage Persistent secretion of lymphatic fluid (≥ 50 ml/24 h) from the surgically inserted drains (lymphatic drainage) or from the wound (lymphorrhea, lymphocutaneous fistula) for more than 5 days or, after drainage removal, postoperative fluid accumulation within the cavity of resection provided the absence of wound dehiscence (lymphocele, lymphocyst, seroma) limiting the adherence of the wound surfaces.		
Grade	A	Persistent lymphatic leakage >5 postoperative days, < 10 postoperative days. Absence of other wound complications
	B	Persistent lymphatic leakage ≥ 10 postoperative days or lymphoceles requiring interventions
	C	Lymphatic leakage leading to reoperation or subsequent conflict with medical measures^a^ or return to normal life^b^
	0	No lymphocele

^a^For example, delay of planned adjuvant treatment (yes/no, delay by × days)

^b^Assessment by the Reintegration to Normal Living (RNL) Index proposed

### Prediction and management of lymphatic leakage

The early diagnosis of a potentially problematic wound situation is relevant to both patients and treating physicians in clinical practice. Our results clearly show a cutoff value on the second postoperative as an indicator for complicated lymphatic leakage (i.e., grades B or C). Multivariate analysis identified the parameters gender, iliac lymph node resection, and resection of the great saphenous vein as risk factors the incidence of complicated lymphatic leakage. These patients have to be monitored closely, and drains should not be removed if the amount of leakage exceeds the threshold. Particularly in grade C lymphatic leakage, early and aggressive therapeutic measures are required in order to allow patients to return to normal life as early as possible—in contrast to grade A patients. According to Lv et al. [9], indications to drain a seroma percutaneously could be impending wound dehiscence, compression of vital structures, or severe discomfort/pain. Surgical reinterventions are necessary in case of wound dehiscence, abscess, hematoma, or development of phlegmon.

### Suggestions for drainage management

In our study, there was great variation in the duration of drainage, stressing the uncertainty in drainage management mainly owing to the lack of a standardized definition of lymphatic leakage and drain removal criteria. There is a broad variation of drain removal criteria in published trials [4, 25, 26]. We propose a removal of suction drains 5 days after surgery if drainage output is less than 50 ml/24 h (no lymphatic leakage). The drains should be removed at least 10 days after surgery in the case of a grade A lymphatic leakage. In contrast to that, if there are early signs indicating a complicated (grade B and C) lymphatic leakage, we assume that a prolonged drainage therapy or the reinsertion of a drain is necessary to prevent wound breakdown.

### Limitations

Further trials involving different centers are necessary to validate the proposed definition and grading of lymphatic leakage prospectively and to prove their clinical applicability and precision.

The validity of the results of this study is subject to the typical limitations of a retrospective design. Missing values—such as a lack of recorded drainage volumes, intercenter differences in the reading of drainage outputs, patient dropout, or a lack of knowledge of reoperations due to treatment of complications in other hospitals—can lead to information or attrition bias. Furthermore, drainage volumes are dependent on technical aspects of surgery and the extent of radicality, which was not explicitly assessed in this study. A larger and prospective acquisition of data would be needed in further trials in order to minimize this kind of bias. However, retrospective data is the basis for prospective evaluations. Our study population is fairly large in comparison to times with adjuvant RILND for any melanoma, and our retrospective data concerning the parameters of the definition is almost complete. It is sufficient to detect significant intergroup differences in median postoperative daily drainage volumes and define a cutoff for the prediction of a lymphatic leakage. We therefore assume that the estimated risk of bias for the definition is rather low.

Even though mainly melanoma patients are included here, to our opinion, the definition does not need to be adapted to different tumor types because RILND is a very well-standardized procedure.

### Future perspectives

If this definition of postoperative lymphatic leakage is used consistently in further studies, the complication rate of RILND will become comparable between different trials. This will in turn make it possible to detect those surgical advances that can lead to a reduction in perioperative morbidity, ultimately improving the oncological outcome for patients. Furthermore, risk factors predisposing to the development of complicated lymphatic leakage after RILND can be detected and compared in future studies. We would also like to lay the basis for future research on whether complicated grade C lymphatic leakage can be prevented by applying the suggested criteria for drainage management or is inevitable.

## Conclusion

Supported by this retrospective analysis of 82 patients, we propose a specific definition for the presence of a postoperative lymphatic leakage after RILND, which represents the first attempt to standardize the diagnosis of this complication. A drainage output of more than 110 ml/24 h on the second postoperative day can serve as a good indicator for the future occurrence of a complicated lymphatic leakage (i.e., grades B or C). This definition might improve our predictive power to identify and compare the postoperative outcome after RILND and might thus be a tool for better clinical decision-making.
